# 

**Published:** 1863-02

**Authors:** 


### ILLINOIS STATE MEDICAL SOCIETY.

The regular annual meeting of the Illinois State Medical Society will be held at Jacksonville, on the first Tuesday in May next, commencing at 10 o'clock in the morning. We hope the profession in every part of the State will be represented, as the meeting will be an important and interesting one. No further postponement will be made on account of the continuance of the rebellion.

N. S. Davis,

*Permanent Secretary of the Ill. State Med. Society.*

Chicago, Ill., Feb. 13th, 1863.

